# Predictive value of combining urinary N-acetyl-β-D-glucosaminidase and serum homocysteine for contrast-induced nephropathy in patients after percutaneous coronary intervention

**DOI:** 10.3389/fcvm.2024.1423836

**Published:** 2024-08-20

**Authors:** Yiling Zhai, Changjun Luo, Nianying Qin, Hongying Cao, Chunyang Dong, Zhou Huang, Dongling Huang, Fan Wang, Wanxia Wei, Jincheng Li, Jie Yang, Xueling Lu, Zhengzhuang Huang, Wei Wang

**Affiliations:** ^1^ Department of Emergency, The First Affiliated Hospital of Guangxi Medical University, Nanning, Guangxi, China; ^2^ Department of Emergency, Affiliated Liutie Central Hospital of Guangxi Medical University, Liuzhou, Guangxi, China; ^3^ Guangxi University Key Laboratory of Emergency Medicine, The First Affiliated Hospital of Guangxi Medical University, Nanning, Guangxi, China; ^4^ Key Laboratory of Molecular Diagnosis and Application, Affiliated Liutie Central Hospital of Guangxi Medical University, Liuzhou, Guangxi, China; ^5^ Department of Cardiovascular Medicine, Affiliated Liutie Central Hospital of Guangxi Medical University, Liuzhou, Guangxi, China; ^6^ Department of Emergency, The First People’s Hospital of Nanning, Nanning, Guangxi, China

**Keywords:** N-acetyl-β-D-glucosaminidase, homocysteine, biomarker, percutaneous coronary intervention, contrast-induced nephropathy

## Abstract

**Background:**

Contrast-induced nephropathy (CIN) can lead to serious complications following percutaneous coronary intervention (PCI). Urine N-Acetyl-β-D-glucosaminidase (uNAG) and serum homocysteine (sHCY) are both potential predictors for CIN detection, but their combination has not been explored. We aimed to combine uNAG and sHCY as predictors for the early detection of CIN and for prognosis prediction in patients after PCI.

**Methods:**

A total of 232 consecutive patients who underwent PCI at a university hospital were recruited for this study. According to the European Society of Urology and Reproduction (ESUR) criterion, CIN is defined as an elevation of serum creatinine (sCr) by ≥25% or ≥0.5 mg/dl from baseline within 48 h. We assessed the use of individual biomarkers (uNAG and sHCY) measured around PCI and their combinations for CIN detection and prognosis prediction. Receiver operating characteristic curves (ROC) and area under the curve (AUC) were used to evaluate the predictive efficiency of potential predictors.

**Results:**

In total, 54 (23.28%) patients developed CIN. Concentrations of uNAG and sHCY increased significantly in CIN subjects (*p *< 0.05) than non-CIN. CIN could be predicted by uNAG and sHCY but not by creatinine at an early stage. At pre-PCI, 0, 12, 24, and 48 h after PCI, the AUC-ROC value of uNAG in calculating total CIN was 0.594, 0.603, 0.685, 0.657, and 0.648, respectively. The AUC-ROC value of sHCY in calculating total CIN was 0.685, 0.726, 0.771, 0.755, and 0.821, respectively. The panel of uNAG plus sHCY detected CIN with significantly higher accuracy than either individual biomarker alone and earlier than sCr. For detecting total CIN, this panel yielded AUC-ROCs of 0.693, 0.754, 0.826, 0.796, and 0.844 at pre-PCI, 0, 12, 24, and 48 h after PCI, respectively, which were superior to those of the individual biomarkers. For predicting the incidence of major adverse cardiovascular events (MACE) within 30 days to 12 months, the AUC-ROC values for uNAG and sHCY measured before discharge were 0.637 and 0.826, respectively. The combined panel yielded an AUC-ROC of 0.832. The combined detection did not significantly enhance the predictive capability for MACE in patients with CIN. The CIN group and the non-CIN group showed no significant difference in the Coronary Heart Disease Intensive Care Unit (CCU) stay time, hospital stay time, demand for renal replacement therapy, CCU mortality rate, and in-hospital mortality rate.

**Conclusions:**

The uNAG and sHCY panel demonstrated better sensitivity and specificity for predicting the diagnosis and prognosis of CIN in patients after PCI, earlier than sCr. The combination of these biomarkers revealed a significantly superior discriminative performance for CIN detection and prognosis compared to using uNAG or sHCY alone.

## 1 Introduction

Due to advancements in medical technologies and interventional cardiology, an increasing number of patients with confirmed or suspected coronary artery disease (CAD) are undergoing percutaneous coronary intervention (PCI) in clinical practice. The widespread use of contrast agents during these procedures has emerged as a significant global concern. Contrast-induced nephropathy (CIN), a major serious complication associated with the use of contrast media administration, is considered the third leading cause of iatrogenic acute kidney injury worldwide and accounts for approximately 11%–12% of cases (1). CIN is associated with prolonged hospitalization, adverse long-term clinical outcomes, and a poor prognosis. The pathophysiological mechanisms responsible for this condition are multifaceted, complex, and not completely understood. Moreover, several reports have identified renal ischemic injury, tubular epithelial cell toxicity, and immunologic reactions as contributing factors (2). Infusion of contrast medium exacerbates hypoxia in the renal medulla and increases the production of renal free radicals via post-ischemic oxidative stress (3). For the early identification of CIN and the provision of a warning of the risk of a poor prognosis, it is urgent to find a promising and potent method for the timely prediction of CIN. Therefore, effective predictors for CIN are crucial to mitigate the risk of developing CIN.

Conventionally, CIN is defined as an elevation of serum creatinine (sCr) of ≥25% or ≥0.5 mg/dl (44 μmol/L) from baseline within 48 h after contrast media administration in the absence of an alternative etiology (4). However, the currently accepted diagnostic criterion for CIN is based on sCr, a biomarker that is late and insensitive, suggesting that it is not an ideal indicator for CIN. Since sCr is secreted by renal tubules and is susceptible to various factors, such as gender, muscle mass, and body distribution, its use can result in delays in early diagnosis and treatment intervention (5). Although the current prevention and treatment guidelines of CIN primarily recommend the use of low- or iso-osmolar contrast media, intravenous hydration before the operation, and a reduction in the contrast dose, there is scarce evidence of other effective, proven strategies during the PCI perioperative period. As a result, the incidence of CIN remains high (6). Therefore, more sensitive biomarkers for CIN need to be explored to prevent its occurrence.

Urine N-Acetyl-β-D-glucosaminidase (uNAG) originates from the lysosomes of the proximal tubule cells in the kidney. The assay of urinary NAG provides a sensitive and early predictor of tubular dysfunction triggered by renal disease or nephrotoxic damage (7). uNAG has been proven to be superior to sCr in predicting CIN in numerous studies. Therefore, it is an ideal predictor for CIN detection and could be used to predict poor outcomes in clinical practice (8, 9). Hyperhomocysteinemia is considered to be independently associated with a greater risk of CIN following angiography or angioplasty, as indicated by previous studies (10, 11). A viewpoint is that hyperhomocysteinemia induces free radicals and oxidative stress due to the direct toxicity of the contrast media, resulting in cellular apoptosis, renal medullary hypoxia, and endothelial dysfunction. These effects share similarities with the proposed pathophysiological mechanisms of CIN (3, 4). If hyperhomocysteinemia is closely related to CIN, then homocysteine is a potential new biomarker for CIN. Early identification of CIN is vital for guiding management strategies. However, the diagnostic accuracy of the combination of uNAG and serum homocysteine (sHCY) for CIN after PCI remains unclear. We hypothesize that this combination would be more valuable in CIN diagnosis than a single indicator after contrast agent exposure. To test our hypothesis, we conducted a prospective, observational study in an adult cardiovascular medicine ward, aiming to assess the performance of combinations of these biomarkers for CIN prediction in patients undergoing PCI.

## 2 Materials and methods

### 2.1 Protocol design and study population

The Cardiovascular Medicine Ward of a tertiary hospital was included in this prospective observational study. From 1 December 2019 to 31 January 2021, consecutive inpatients receiving PCI for any reason were enrolled. Exclusion criteria included refusal of consent, age <18 years, pregnancy, unavailability of urine samples, exposure to nephrotoxic drugs prior to or during the study period, hemodialysis or peritoneal dialysis prior to enrollment or end-stage renal disease (ESRD), nephrectomy or renal transplantation, impaired renal function [estimated glomerular filtration rate (eGFR) <60 ml/min/1.73 m^2^], contraindication for β-blockers, and allergy to iodine-containing contrast medium. The protocol followed the Strengthening the Reporting of Observational Studies in Epidemiology (STROBE) and Standards for Reporting Diagnostic Accuracy (STARD) criteria (12, 13). The current study received the approval from the local institutional review board. Written informed consent was obtained from each participant or a family member at the time of enrollment.

### 2.2 Specimen and data collection

Urine and blood samples were collected before PCI. Patients were hydrated with intravenous normal saline (1.0–1.5 ml/kg/h) for 6–12 h before elective PCI. After the procedure, the hydration was continued for 24 h at a rate of 1.0 ml/kg/h, which was decreased to 0.5 ml/kg/h for patients with volume overload status or left ventricular systolic dysfunction (ejection fraction 40%). Emergency PCI was performed for patients with ST-segment elevation myocardial infarction. Next, the same samples were collected at 0h (within 1 h after PCI), 12 h, 24 h, 48 h after PCI, as well as before discharge. For patients from the participating hospitals, urine and blood samples were shipped by cold chain (4℃) transportation and examined at the central laboratory of the Affiliated Liutie Central Hospital of Guangxi Medical University within 2 h using a standard protocol after collection. According to the testing specifications, none of the samples would degrade significantly in short-term storage. We measured the levels of sCr, uNAG, and sHCY around the time of the PCI operation. sCr was measured at admission to the Cardiovascular Medicine Ward and as a part of routine clinical care during the in-hospital stay. We prospectively collected the demographic and clinical characteristics of each patient, including sex, age, body mass index (BMI), preexisting chronic conditions, the categories of diseases, surgery-related information, Global Registry of Acute Coronary Events score (GRACE), Mehran score, baseline sCr, baseline eGFR, and outcomes. The baseline eGFR for patients was calculated using the abbreviated Modification of Diet in Renal Disease formula (14).

### 2.3 Biomarker assays

Following the manufacturer's instructions, sCr, uNAG, and sHCY were analyzed using a biochemical autoanalyzer (HITACHI 3500, HITACHI, Tokyo, Japan). sCr was determined using the oxidase method, with the normal reference range being 40–97 μmol/L for males and 40–80 μmol/L for females. The values of uNAG were measured using a 6-methyl-2-mercaptopyridine (MPT) substrate detection method, with the normal reference range being 0.3–14.6 U/L. The coefficients of interassay and intraassay variation for uNAG were both ≤10%. sHCY was measured using an enzyme recycling method, with the normal reference range being 3.0–15.4 μmol/L. The coefficients of interassay and intraassay variation in sHCY were <5% and ≤10%, respectively. The personnel measuring all the biomarkers were completely blinded to each patient's clinical information and illness characteristics.

### 2.4 Data definitions

The most commonly used CIN definition is based on the criteria of the European Society of Urogenital Radiology (ESUR) (15): sCr levels increased by more than 25% relatively compared with baseline value or by 0.5 mg/dl (44 μmol/L) absolutely from baseline within 48–72 h after a diagnostic or interventional procedure, in the absence of an alternative etiology. CIN assessment is classified into three stages: stage 0, stage 1, and stage 2. Stage 0: a relative increase of <25% in sCr from baseline and an absolute increase of <0.5 mg/dl; stage 1: a relative increase of ≥25% in sCr from baseline and an absolute increase of <0.5 mg/dl; stage 2: a relative increase of ≥25% in sCr from baseline and an absolute increase of ≥0.5 mg/dl. Mild CIN was defined as ESUR stage 1 and severe CIN as ESUR stage 2 after contrast agent exposure.

The baseline sCr was defined using the following rules in sequence as described in a previous study (16). If patients had a serum creatinine value before inpatient admission: (1) the most recent pre-inpatient value (between 30 and 365 days before inpatient admission); (2) for patients aged <40 years, a stable pre-inpatient value >365 days before admission (stable defined as within 15% of the lowest inpatient measurement); (3) pre-inpatient value (>365 days before admission) and less than the initial sCr at admission; (4) a pre-inpatient value (between 3 and 39 days before admission) ≤ initial sCr at the time of admission to ward and not distinctly in CIN; if patients did not have serum creatinine value before admission; (5) the lowest sCr value upon initial admission, the final ward value, or the minimum value at follow-up up to 365 days.

Major adverse cardiovascular events (MACEs) primarily included non-fatal myocardial infarction, non-fatal stroke, cardiogenic shock, various arrhythmia, late revascularization, heart failure (HF) rehospitalization, and all-cause mortality.

### 2.5 Statistical analysis

Statistical analysis was performed using SPSS 24.0 (SPSS Inc., Chicago, IL, USA) and GraphPad Prism 8.4.3 (GraphPad Software Inc., San Diego, CA, USA) software. For normally distributed data, continuous variables were presented as means ± standard deviation (SD). For comparisons between the CIN and non-CIN groups, the independent-sample *t*-test was used. The distribution of skew data was expressed as M (P25–P75), and the comparison between groups was conducted by non-parametric test. Categorical variables were expressed as percentages, and compared using the chi-squared test. All the tests were two-tailed, and *p* <0.05 was considered to be statistically significant unless otherwise specified. The software program MedCalc version 20.110 (MedCalc Software, Ostend, Belgium) was utilized to measure receiver operating characteristic (ROC) curves with their area under the curve (AUCs) drawn to detect the optimum cut-offs of parameters and their sensitivity and specificity in predicting CIN. The optimum cutoff is defined as the point where one gets a maximum value of Youden's index (J = sensitivity + specificity − 1) in the ROC curve. The sensitivity, specificity, positive and negative predictive values (PPV and NPV, respectively), and positive and negative likelihood ratios ([+]LR and [−]LR, respectively) of the biomarkers were calculated as well.

## 3 Results

### 3.1 Patient characteristics

**Figure 1:**
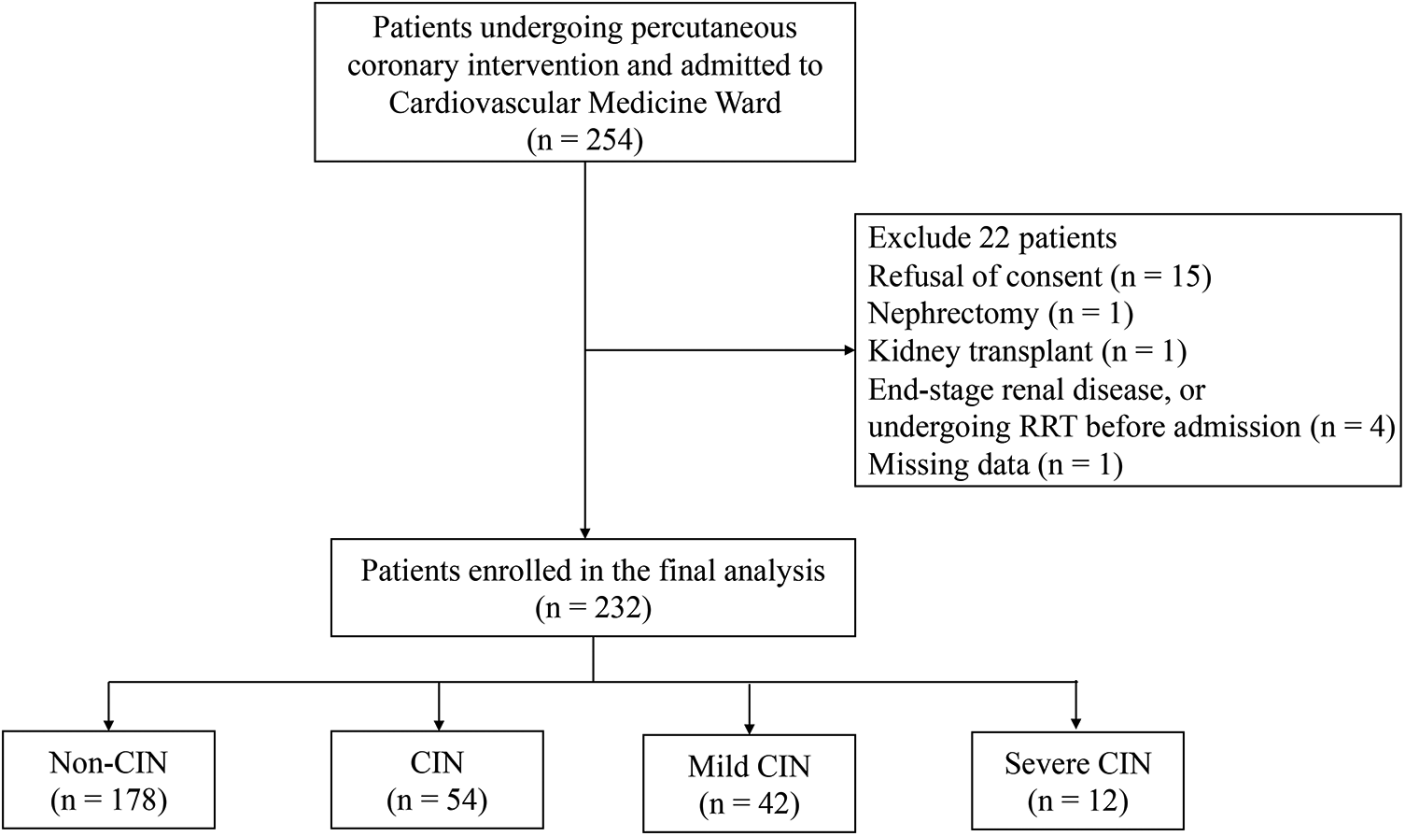
Patient screening flow chart. CIN, contrast-induced nephropathy.

Of the 254 consecutive adult patients screened for inclusion in the study (Figure 1), 22 (8.7%) were excluded for the following reasons: refusal to consent (*N* = 15), nephrectomy (*N* = 1), kidney transplant (*N* = 1), missing admission data (*N* = 1), ESRD, or undergoing renal replacement therapy (RRT) before cardiovascular medicine ward admission (*N* = 4). Therefore, a total of 232 (91.3%) eligible patients were enrolled in the analysis. According to the definition of CIN, which relies on the changes in sCr levels within 48–72 h after PCI, patients were assigned to either CIN(+) or CIN(−) group. In total, 54 (23.28%) of the patients developed CIN after the infusion of contrast medium, while 178 (76.72%) did not.

Patient characteristics are shown in Table 1. Compared with the non-CIN patients, patients with CIN(+) had a higher rate of preexisting clinical conditions, such as renal insufficiency (11.1% vs. 2.2%, *p* = 0.015) and cerebrovascular disease (35.2% vs. 20.8%, *p* = 0.047). Among patients who developed CIN, their preoperative GRACE score and systolic blood pressure were significantly higher than those of the CIN(−) group (*p* = 0.038 and 0.037). In preoperative medication history, patients who had taken beta-blockers had a lower incidence rate of CIN. However, the other baseline profiles and clinic outcomes showed no significant difference between the CIN(+) and CIN(−) groups.

**Table 1:** Baseline characteristics and outcomes (*N* = number of individual).

Characteristics	Non-CIN group (*N* = 178)		CIN group (*N* = 54)		*p*-value
Demographic variables	Mean	SD	Mean	SD	
Age (years)	63.20	10.90	63.90	10.20	0.671
BMI (kg/m^2^)	24.60	2.96	25.00	3.17	0.371
Pulse (bpm)	72.70	13.90	74.70	14.50	0.380
Systolic blood pressure (mmHg)	133.00	23.30	141.00	24.40	**0.037**
Diastolic blood pressure (mmHg)	80.90	15.00	82.40	15.40	0.521
Demographic variables	Frequency	%	Frequency	%	
Male gender, *N* (%)	128	71.9	46	85.20	0.073
Smoker, *N* (%)	61	34.30	23	42.60	0.341
Drinker, *N* (%)	28	15.70	8	14.80	1.000
Preexisting clinical conditions	Frequency	%	Frequency	%	
DM, *N* (%)	50	28.10	17	31.50	0.756
Hypertension, *N* (%)	104	58.40	38	70.40	0.150
HF, *N* (%)	86	46.10	23	42.60	0.769
Cancer, *N* (%)	3	1.70	1	1.90	1.000
Thyroid disease, *N* (%)	8	4.50	6	11.10	0.144
COPD, *N* (%)	0	0	1	1.90	0.526
Cerebrovascular disease, *N* (%)	37	20.80	19	35.20	**0.047**
Digestive disease, *N* (%)	36	20.20	13	24.10	0.677
Arrhythmia, *N* (%)	12	6.70	4	7.40	1.000
Renal insufficiency, *N* (%)	4	2.20	6	11.10	**0.015**
Malignancy, *N* (%)	3	1.70	1	1.90	1.000
Preoperative medication history	Frequency	%	Frequency	%	
ACEI/ARB, *N* (%)	49	27.50	11	20.40	0.382
CCB, *N* (%)	34	19.10	13	24.10	0.546
β-blockers, *N* (%)	25	14.0	1	1.90	**0.025**
Statin, *N* (%)	21	11.80	3	5.60	0.287
Diuretic, *N* (%)	5	2.80	1	1.90	1.000
Uric acid control drugs, *N* (%)	1	0.60	2	3.70	0.270
Hypoglycemic agents, *N* (%)	34	19.10	14	25.90	0.372
Baseline information	Mean	SD	Mean	SD	
Baseline serum creatinine (μmol/L)	74.20	21.40	74.80	27.00	0.891
Baseline eGFR (ml/minute/1.73 m^2^)	88.39	16.89	89.69	21.25	0.643
GRACE score	119.00	30.50	128.00	27.00	**0.038**
Mehran score	3.48	3.39	3.46	3.32	0.977
Outcomes	Mean	SD	Mean	SD	
Length of CCU stay (days)	2.17	3.25	2.41	2.78	0.596
Length of hospital stay (days)	7.28	3.12	7.70	2.77	0.337
Outcomes	Frequency	%	Frequency	%	
RRT during CCU stay, *N* (%)	0	0	1	1.85	0.526
CCU mortality, *N* (%)	0	0	1	1.85	0.526
In-hospital mortality, *N* (%)	0	0	2	3.70	0.082

CIN, contrast-induced nephropathy; BMI, body mass index; DM, diabetes mellitus; HF, heart failure; COPD, chronic obstructive pulmonary disease; ACEI, angiotensin-converting enzyme inhibitor I; ARB, angiotensin II receptor antagonist; CCB, calcium channel blocker; eGFR, estimated glomerular filtration rate; sCr, serum creatinine; GRACE, Global Registry of Acute Cardiovascular Events; RRT, renal replacement therapy; CCU, coronary heart disease intensive care unit.

Categorical variables are expressed as number (%). Bold values indicate significant *p*-values <0.05.

Biochemical parameters were detected after PCI. Patients with CIN(+) had significantly higher levels of biochemical indexes than non-CIN patients, including C-reactive protein (CRP), total cholesterol (CHO), and low-density lipoprotein (LDL-C) (*p *< 0.05, Table 2).

**Table 2:** Biochemical parameters characteristics (*N* = number of individual).

Biochemical parameters	Non-CIN group (*N* = 178)		CIN group (*N* = 54)		*p*-value
Baseline biochemical parameters	Mean	SD	Mean	SD	
NT-proBNP (pg/ml)	3,440	1,470	3,210	1,460	0.679
Hb (g/L)	137.00	19.00	133.00	20.40	0.227
HCT (%)	41.00	5.21	40.00	5.72	0.279
RDW-CV (%)	13.10	1.15	13.00	0.82	0.901
RDW-SD (fl)	42.20	3.67	42.30	3.15	0.766
CRP (mg/L)	11.70	2.60	14.80	8.00	0.453
Hs-CRP (mg/L)	7.91	3.10	11.60	4.80	0.115
ALB (g/L)	38.60	4.39	37.70	5.20	0.222
CHO (mmol/L)	4.61	1.17	4.97	1.52	0.117
TC (mmol/L)	2.11	1.26	2.18	1.87	0.814
HDL-C (mmol/L)	1.03	0.24	0.98	14.80	0.181
LDL-C (mmol/L)	2.76	0.97	2.82	0.25	0.690
Lpa (mg/L)	267.00	82.00	260.00	27.00	0.886
HbA1c (%)	6.69	1.63	6.81	2.09	0.703
T3 (nmol/L)	0.91	0.22	0.92	0.80	0.958
T4 (nmol/L)	69.50	14.30	68.4	21.30	0.704
FT3 (pmol/L)	4.04	0.85	4.56	0.36	.554
FT4 (pmol/L)	15.80	5.40	16.50	9.34	0.632
TSH (uIU/ml)	2.23	1.66	3.79	1.50	0.328
Biochemical parameters after PCI	Mean	SD	Mean	SD	
NT-proBNP (pg/ml)	1,790	1,220	1,810	1,380	0.963
Hb (g/L)	130.00	17.90	133.00	17.60	0.243
HCT (%)	39.10	4.93	40.00	4.97	0.219
RDW-CV (%)	13.10	1.08	13.10	0.83	0.826
RDW-SD (fL)	41.80	3.50	42.30	2.94	0.369
CRP (mg/L)	18.10	4.10	31.80	6.00	**0** **.** **040**
Hs-CRP (mg/L)	12.70	8.50	18.30	5.70	0.147
ALB (g/L)	36.90	4.38	36.20	3.46	0.214
CHO (mmol/L)	4.24	1.23	4.70	1.24	**0** **.** **019**
TC (mmol/L)	1.67	0.93	1.82	0.38	0.455
HDL-C (mmol/L)	1.02	0.25	1.06	0.21	0.331
LDL-C (mmol/L)	2.51	0.99	2.92	1.10	**0** **.** **016**
Lpa (mg/L)	248.00	40.00	265.00	78.00	0.695

CIN, contrast-induced nephropathy; NT-proBNP, N-terminal pro B-type natriuretic peptide; Hb, hemoglobin; HCT, hematocrit; RDW-CV, variation coefficient of erythrocyte volume distribution width; RDW-SD, standard deviation of erythrocyte distribution width; CRP, c-reactive protein; Hs-CRP, hypersensitive c-reactive protein; ALB, albumin; CHO, total cholesterol; TC, triglyceride; HDL-C, high-density lipoprotein; LDL-C, low-density lipoprotein; Lpa, lipoprotein a; HbA1c, glycosylated hemoglobin; T3, triiodothyronine; T4, thyroxine; FT3, free triiodothyronine; FT4, free thyroxine; TSH thyroid stimulating hormone.

Categorical variables are expressed as number (%). Bold values indicate significant *p*-values <0.05.

Surgery-related information is demonstrated in Table 3. Patients with ST-segment elevation myocardial infarction (STEMI) had a higher portion of CIN (*p* = 0.049) than those with other cardiovascular diseases, such as non-ST-segment elevation myocardial infarction (NSTEMI), acute coronary syndrome (ACS), unstable angina (UA), CAD. Longer surgery durations and greater total lengths of heart stents were observed in patients with CIN.

**Table 3:** Surgery-related information (*N* = number of individual).

Characteristics	Non-CIN group (*N* = 178)		CIN group (*N* = 54)		*p*-value
Diagnosis	Frequency	%	Frequency	%	
STEMI, *N* (%)	64	36.00	29	53.70	**0** **.** **049**
NSTEMI, *N* (%)	8	4.50	2	3.70	
ACS, *N* (%)	5	2.80	1	1.90	
UA, *N* (%)	30	16.90	12	22.20	
CAD, *N* (%)	71	39.90	10	18.50	
Surgery-related information	Frequency	%	Frequency	%	
Surgery history, *N* (%)	38	21.30	11	20.40	1.000
PCI history, *N* (%)	38	21.30	9	16.70	0.578
Surgery-related information	Mean	SD	Mean	SD	
Surgery duration (minute)	66.90	26.00	78.20	38.10	**0** **.** **046**
Number of coronary artery lesions, *N*	2.03	0.83	2.07	0.80	0.748
Number of stents, *N*	1.48	0.64	1.61	0.60	0.160
Total length of stents (mm)	37.50	19.90	44.00	21.30	**0** **.** **049**
Dose of contrast medium (ml)	109.00	28.50	113.00	32.00	0.439
Intraoperative medication	Frequency	%	Frequency	%	
Prourokinase, *N* (%)	11	6.20	6	11.10	0.358
Vasoactive drugs, *N* (%)	66	37.10	20	37.00	1.000
Tirofiban, *N* (%)	59	33.10	21	38.90	0.539
Types of contrast medium	Frequency	%	Frequency	%	
Ioversol, *N* (%)	121	68.00	37	68.50	0.981
Iodixanol, *N* (%)	48	27.00	14	25.90	0.981
Iohexol, *N* (%)	9	5.10	3	5.60	0.981

CIN, contrast-induced nephropathy; STEMI, ST-segment elevation myocardial infarction; NSTEMI, non-ST-segment elevation myocardial infarction; ACS, acute coronary syndrome; UA, unstable angina; CAD, coronary artery disease; PCI, percutaneous coronary intervention.

Categorical variables are expressed as number (%). Bold values indicate significant *p*-values <0.05.

### 3.2 CIN detection by biomarkers measured during the perioperative period of PCI

Of the 54 patients with CIN, there were 42 cases (18.10%) of mild CIN (ESUR stage 1) and 12 cases (5.17%) of severe CIN (ESUR stage 2). ROC curve analysis revealed that the two biomarkers studied detected total CIN with statistical significance. Patients with CIN had higher concentrations of sHCY during preoperative and postoperative periods of PCI compared to the non-CIN group (*p* < 0.001). After the coronary intervention, the level of uNAG in patients with CIN(+) began to increase at 0 h after PCI and yielded a *p*-value <0.05 compared to that in the CIN(−) group. The levels of uNAG and sHCY in the CIN(+) group were significantly higher than those in the CIN(−) group (*p* < 0.05), and these increases were observed much earlier than the changes in sCr levels. The sCr levels in patients with severe CIN began to increase at 12 h after PCI, which was significantly different compared with those in patients with non-CIN and mild CIN (*p* < 0.05). However, the concentrations of sHCY were not significantly different during the preoperative and postoperative periods of PCI, and there were no significant differences in the levels of uNAG and sHCY between the mild and severe CIN groups (*p* > 0.05, Table 4).

**Table 4:** The biomarkers of different groups at different time (*N* = number of individual).

Biomarkers	Non-CIN group (*N* = 178)		CIN group (*N* = 54)		Mild CIN group (*N* = 42)		Severe CIN group (*N* = 12)	
T1	Mean	SD	Mean	SD	Mean	SD	Mean	SD
uNAG (U/L)	6.45	3.90	6.36	4.19	6.24	4.68	3.35	2.03
sHCY (μmol/L)	13.90	5.77	18.70	9.28^a^	18.10	8.65^a^	16.50	6.44^a^
sCr (μmol/L)	80.3	22.8	83.7	28.3	79.8	23.00	96.40	41.20
T2	Mean	SD	Mean	SD	Mean	SD	Mean	SD
uNAG (U/L)	6.34	4.05	8.66	6.38^b^	8.44	6.56^b^	8.87	6.48^b^
sHCY (μmol/L)	13.50	7.09	19.00	12.00^b^	19.50	13.20^b^	15.30	6.74^b^
sCr (μmol/L)	76.30	24.00	82.50	29.00	78.10	23.60	96.80	42.60
T3	Mean	SD	Mean	SD	Mean	SD	Mean	SD
uNAG (U/L)	6.99	4.10	12.60	10.80^a^	11.30	7.23^a^	17.60	11.30^a^
sHCY (μmol/L)	13.60	5.00)	20.40	11.10^a^	20.80	12.30^a^	18.90	5.06^a^
sCr (μmol/L)	79.80	23.80	90.40	33.30^b^	82.70	26.00^b^	117.00	42.70^b^^,^^c^
T4	Mean	SD	Mean	SD	Mean	SD	Mean	SD
uNAG (U/L)	6.39	4.17	10.90	8.95^a^	9.09	6.17^a^	17.20	13.70^a^
sHCY (μmol/L)	14.20	7.75	20.50	11.10^a^	21.00	12.30^a^	18.90	5.65^a^
sCr (μmol/L)	82.50	23.90	95.90	32.90^b^	88.30	26.10^b^	122.00	41.20^b^^,^^c^
T5	Mean	SD	Mean	SD	Mean	SD	Mean	SD
uNAG (U/L)	5.58	4.10	9.06	7.11^b^	8.25	6.43^b^	11.60	8.01^b^
sHCY (μmol/L)	13.40	4.79	20.50	10.30^a^	20.70	11.20^a^	20.30	6.18^a^
sCr (μmol/L)	82.30	24.40	96.70	34.10^b^	87.20	23.90^b^	132.00	40.70^b^^,^^c^
T6	Mean	SD	Mean	SD	Mean	SD	Mean	SD
uNAG (U/L)	5.03	5.31	8.83	6.40^b^	8.31	5.80^b^	8.72	6.96^b^
sHCY (μmol/L)	13.1	3.84	19.80	9.67^a^	20.3	10.40^a^	19.4	6.46^a^
sCr (μmol/L)	82.40	25.40	101.00	61.90^a^	87.50	25.60^b^	152.00	112.00^b^

CIN, contrast-induced nephropathy; uNAG, urine N-Acetyl-β-D-glucosaminidase; sHCY, serum homocysteine; sCr, serum creatinine; *T1*_,_ pre-PCI, *T2,* within 1 h after PCI; *T3,* 12 h after PCI; *T4,* 24 h after PCI; *T5,* 48 h after PCI; *T6*, before discharge.

^a^ Compared with the non-CIN group indicate significant *p*-values <0.001.

^b^ Compared with the non-CIN group indicate significant *p*-values <0.05.

^c^ Compared with the Mild CIN group indicate significant *p*-values <0.05.

### 3.3 Predictive abilities of single biomarker and their combinations in CIN detection

uNAG detected total CIN with a sensitivity of 71.4% and a specificity of 50.0% at pre-PCI (*T1*), a sensitivity of 85.4% and a specificity of 33.3% at within 1 h after PCI (*T2*), a sensitivity of 73.6% and a specificity of 63.0% at 12 h after PCI (*T3*), a sensitivity of 80.9% and a specificity of 42.6% at 24 h after PCI (*T4*), and a sensitivity of 51.7% and a specificity of 74.1% at 48 h after PCI (*T5*), respectively. uNAG identified total CIN with the highest sensitivity immediately after PCI (*T2*) and the highest specificity at 48 h after PCI (*T5*). sHCY detected total CIN with a sensitivity of 90.5% and a specificity of 44.4% at *T1*, a sensitivity of 82.0% and a specificity of 61.1% at *T2*, a sensitivity of 78.7% and a specificity of 66.7% at *T3*, a sensitivity of 91.0% and a specificity of 55.6% at *T4*, and a sensitivity of 92.7% and a specificity of 59.3% at *T5*, respectively. sHCY identified total CIN with the highest sensitivity at 48 h after PCI (*T5*) and the highest specificity at 12 h after PCI (*T3*) (Table 5).

**Table 5:** Predictive characteristics of biomarkers and their combinations for total CIN at different time.

Biomarkers	AUC-ROC	95% CI	Cut-off^a^	Sensitivity	Specificity	(+)LR	(−)LR	PPV	NPV
T1									
uNAG	0.594	0.502–0.685	4.00	0.714	0.500	1.427	0.573	0.305	0.856
sHCY	0.685	0.596–0.774	18.15	0.905	0.444	1.628	0.214	0.331	0.940
uNAG + sHCY	0.693	0.602–0.785		0.815	0.556	1.836	0.333	0.358	0.908
T2									
uNAG	0.603	0.516–0.690	9.90	0.854	0.333	1.280	0.438	0.279	0.881
sHCY	0.726	0.637–0.815	15.90	0.820	0.611	2.108	0.295	0.389	0.916
uNAG + sHCY	0.754	0.675–0.833		0.770	0.685	2.444	0.336	0.429	0.910
T3									
uNAG	0.685	0.591–0.779	8.65	0.736	0.630	1.989	0.419	0.377	0.889
sHCY	0.771	0.693–0.848	15.95	0.787	0.667	0.363	0.319	0.419	0.908
uNAG + sHCY	0.826	0.758–0.894		0.927	0.611	2.383	0.119	0.420	0.965
T4									
uNAG	0.657	0.571–0.742	9.75	0.809	0.426	1.409	0.448	0.301	0.884
sHCY	0.755	0.675–0.834	18.05	0.910	0.556	2.050	0.162	0.383	0.952
uNAG + sHCY	0.796	0.730–0.862		0.719	0.796	3.525	0.353	0.520	0.904
T5									
uNAG	0.648	0.565–0.731	5.15	0.517	0.741	1.996	0.652	0.378	0.835
sHCY	0.821	0.753–0.888	17.50	0.927	0.593	2.278	0.123	0.410	0.964
uNAG + sHCY	0.844	0.777–0.910		0.882	0.741	3.405	0.159	0.511	0.957

ROC, receiver operating characteristic curves; AUC, area under the curve; CI, confidence interval; PPV, positive predictive values; NPV, negative predictive values; [+]LR, positive likelihood ratios; [−]LR, negative likelihood ratios; uNAG, urine N-Acetyl-β-D-glucosaminidase; sHCY, serum homocysteine; *T1,* pre-PCI, *T2*, within 1 h after PCI; *T3,* 12 h after PCI; *T4*, 24 h after PCI; *T5,* 48 h after PCI.

^a^ Ideal cutoff value according to Youden's index.

**Table 6:** Predictive characteristics of biomarkers and their combinations before discharge for MACE (*T6*).

Biomarkers	AUC-ROC	95% CI	Cut-off^a^	Sensitivity	Specificity	(+)LR	(−)LR	PPV	NPV
T6									
uNAG	0.637	0.557–0.724	7.25	0.815	0.407	1.374	0.455	0.295	0.880
sHCY	0.826	0.764–0.888	15.30	0.809	0.704	2.730	0.271	0.454	0.926
uNAG + sHCY	0.832	0.771–0.893		0.899	0.630	2.427	0.161	0.426	0.957

ROC, receiver operating characteristic curves; AUC, area under the curve; CI, confidence interval; PPV, positive predictive values; NPV, negative predictive values; [+]LR, positive likelihood ratios; [−]LR, negative likelihood ratios; uNAG, urine N-Acetyl-β-D-glucosaminidase; sHCY, serum homocysteine; *T6*, before discharge.

^a^ Ideal cutoff value according to Youden's index.

To improve the performance of these biomarkers in CIN detection, we developed possible panels consisting of these two biomarkers (uNAG + sHCY). The panel of uNAG plus sHCY detected total CIN at 12 h after PCI (*T3*) with relatively high sensitivity (92.7%) but low specificity (61.1%). Furthermore, the panel of uNAG plus sHCY detected total CIN at 24 h after PCI (*T4*) with relatively high specificity (79.6%) but limited sensitivity (71.9%). Both panels demonstrated better performance than the individual biomarkers (Table 5).

**Figure 2:**
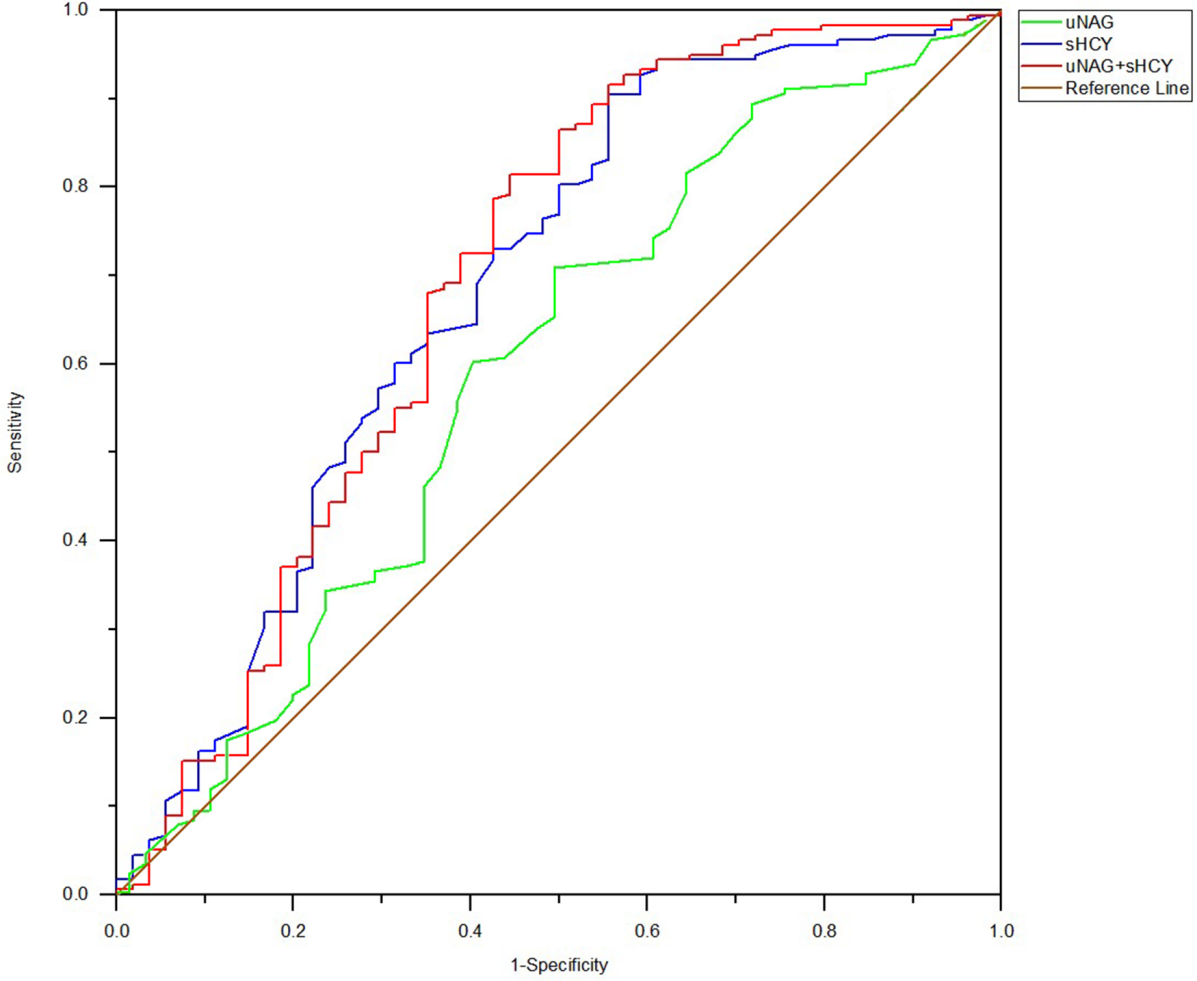
ROC analysis of uNAG plus sHCY and their combinations for CIN detection at pre-PCI (*T1*). AUC-ROC (95% CI): uNAG 0.594 (0.502–0.685), sHCY 0.685 (0.596–0.774), and uNAG + sHCY 0.693 (0.602–0.785).

**Figure 3:**
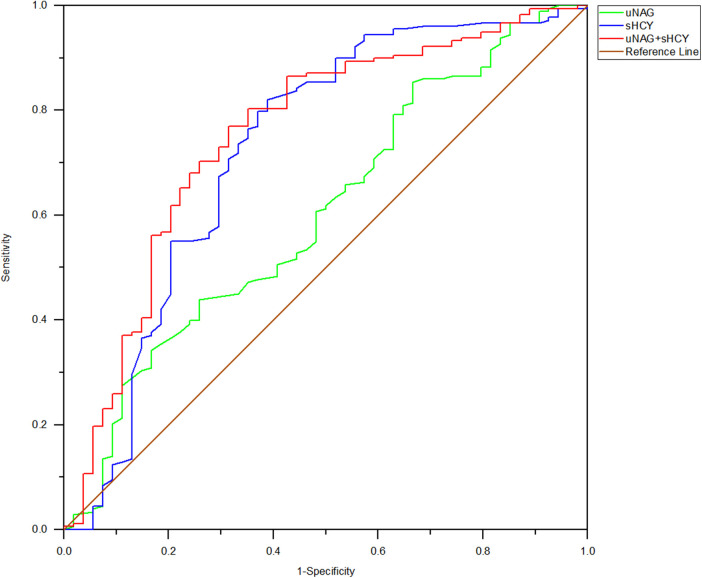
ROC analysis of uNAG plus sHCY and their combinations for CIN detection at 0 h after PCI (*T2*). AUC-ROC (95% CI): uNAG 0.603 (0.516–0.690), sHCY 0.726 (0.637–0.815), and uNAG + sHCY 0.754 (0.675–0.833).

**Figure 4:**
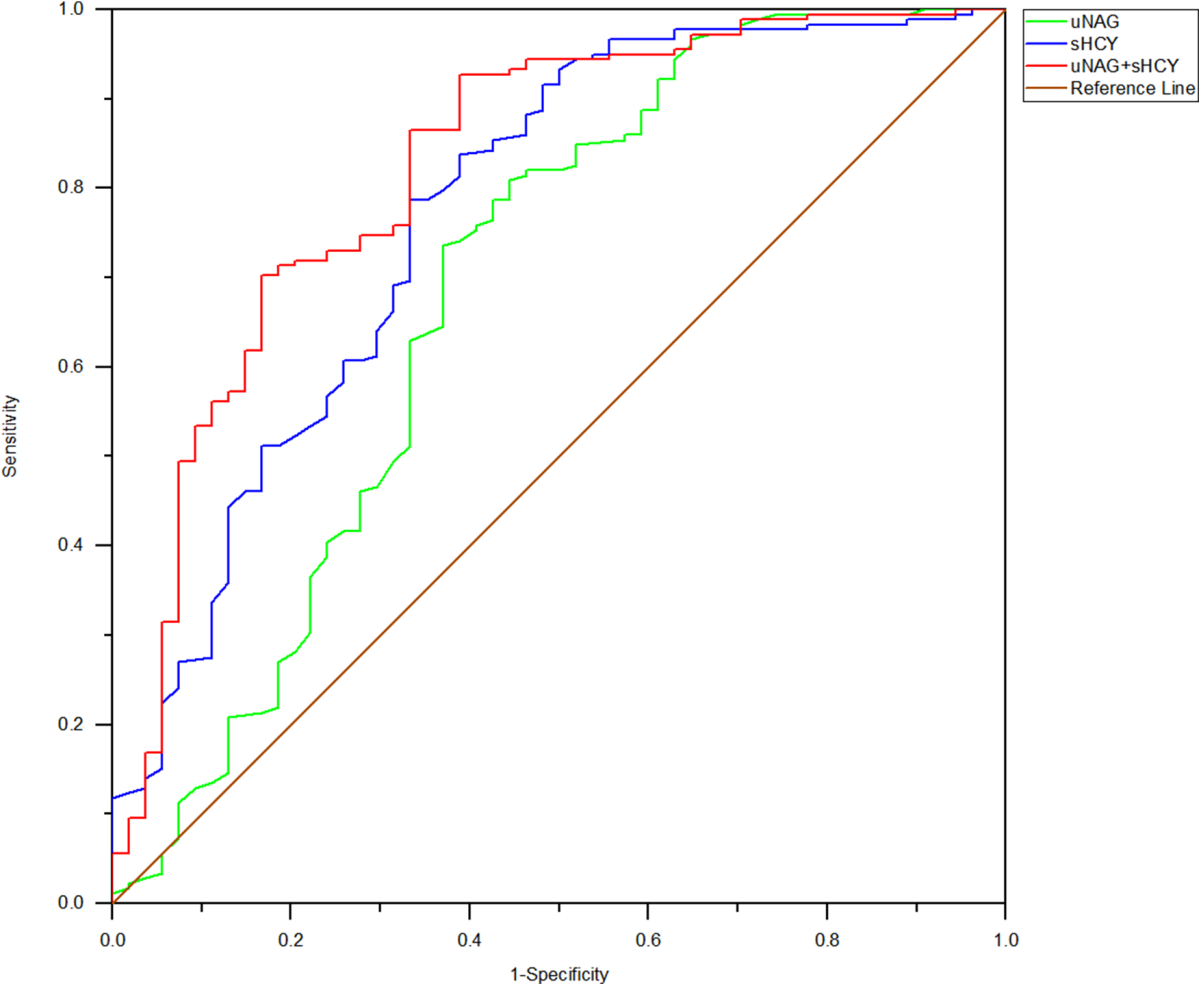
ROC analysis of uNAG plus sHCY and their combinations for CIN detection at 12 h after PCI (*T3*). AUC-ROC (95% CI): uNAG 0.685 (0.591–0.779), sHCY 0.771 (0.693–0.848), and uNAG + sHCY 0.826 (0.758–0.894).

**Figure 5:**
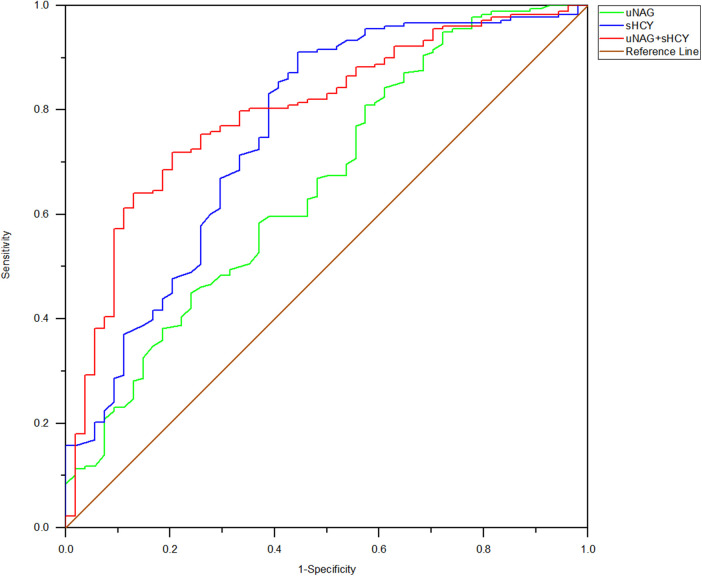
ROC analysis of uNAG plus sHCY and their combinations for CIN detection at 24 h after PCI (*T4*). AUC-ROC (95% CI): uNAG 0.657 (0.571–0.742), sHCY 0.755 (0.675–0.834), and uNAG + sHCY 0.796 (0.730–0.862).

**Figure 6:**
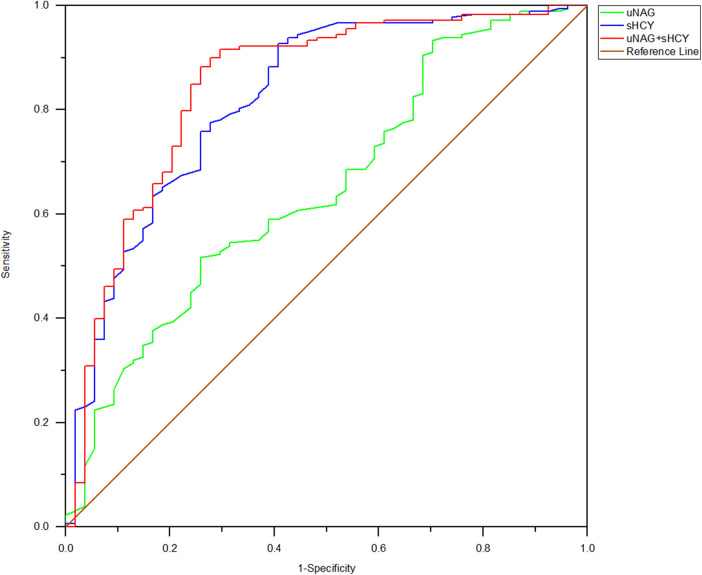
ROC analysis of uNAG plus sHCY and their combinations for CIN detection at 48 h after PCI (*T5*). AUC-ROC (95% CI): uNAG 0.648 (0.565–0.731), sHCY 0.821 (0.753–0.888), and uNAG + sHCY 0.844 (0.777–0.910).

In the entire cohort, the predictive abilities of the biomarker combinations for CIN detection were assessed. The AUC-ROCs for total CIN demonstrated better performance of the panel of uNAG plus sHCY than a single biomarker at *T1, T2, T3, T4,* and *T5*, respectively. The combination of the two biomarkers demonstrated poor to moderate AUC-ROC values for predicting CIN (0.693 and 0.754, respectively) at pre-PCI (*T1*) and immediately after PCI (*T2*). However, the panel of uNAG plus sHCY had higher AUC-ROC values for the prediction of CIN at 12 h (*T3*), 24 h (*T4*), and 48 h (*T5*) after PCI (0.826, 0.796, and 0.844, respectively). The panel's highest AUC-ROC value for CIN detection was 0.844. We concluded these biomarker combinations had the highest predictive abilities at 48 h after PCI (*T5*) compared to other time points (Table 5; Figures 2–6). The analysis demonstrated very similar results to the main analysis shown in Table 4.

When Youden's index was at its maximum, the best diagnostic thresholds for predicting CIN by uNAG were 4.00, 9.90, 8.65, 9.75, and 5.15 U/L, respectively. The best diagnostic thresholds for CIN prediction by sHCY were 18.15, 15.90, 15.95, 18.05, and 17.50 μmol/L, respectively (Table 5).

### 3.4 Prediction of MACE incidence rate by biomarkers measured before discharge

**Figure 7:**
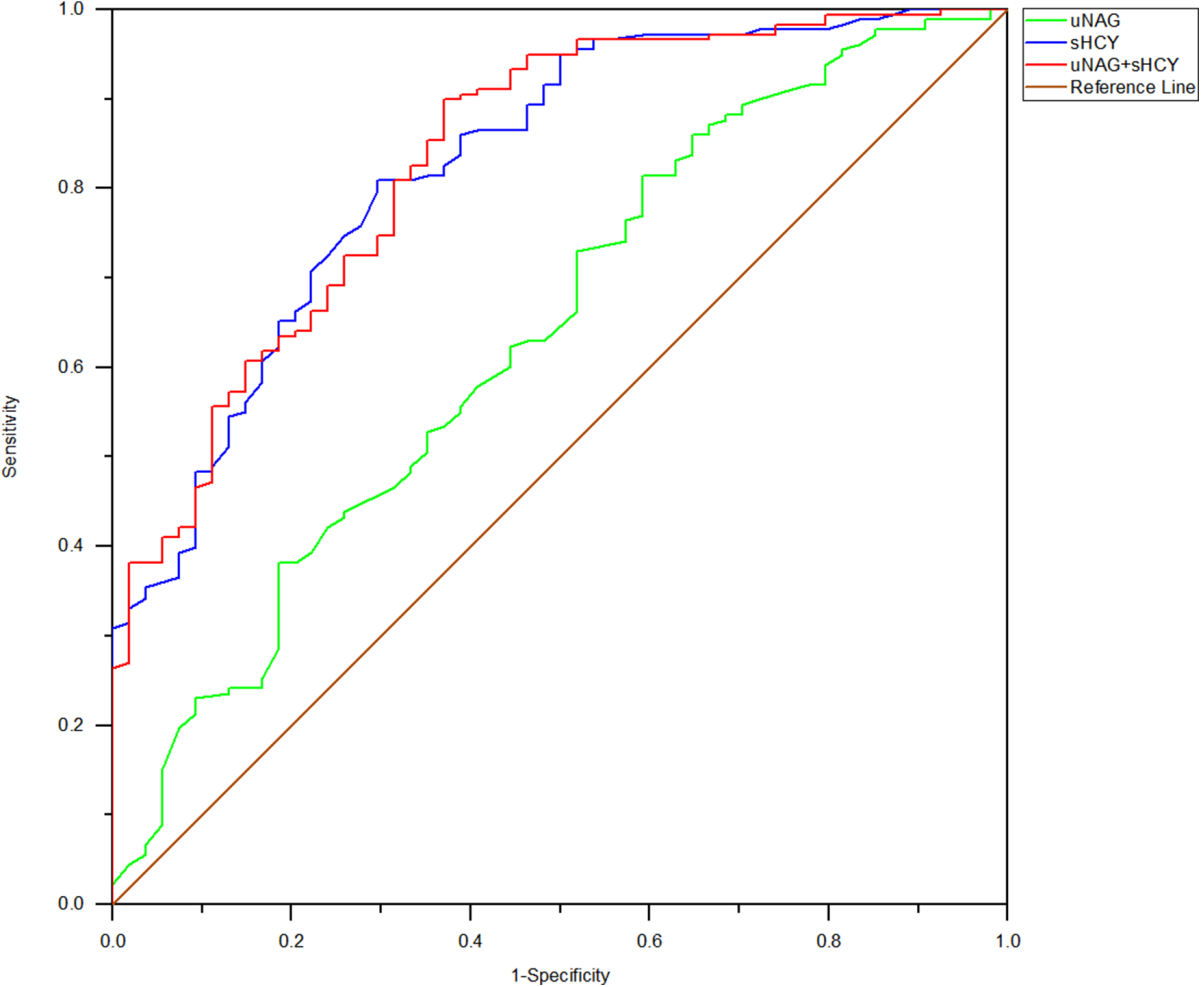
ROC analysis of uNAG plus sHCY and their combinations measured before discharge (*T6*) for predicting MACE within 30 days to 12 months. AUC-ROC (95% CI): uNAG 0.637 (0.557–0.724), sHCY 0.826 (0.764–0.888), and uNAG + sHCY 0.832 (0.771–0.893).

Among the 232 adult ill patients, 1 died in the Coronary Heart Disease Intensive Care Unit (CCU) (0.431%) and 2 died during their in-hospital stay (0.862%). Prognostic information about the remaining 230 patients was comprehensively tracked from 30 days to 12 months after discharge. During the follow-up, 15 (6.47%) cumulative MACE incidents were documented. Of the 52 patients with CIN(+), 13 suffered a MACE, which included 1 (0.43%) case of non-fatal myocardial infarction, 1 (0.43%) case of non-fatal stroke, 1 (0.43%) case of cardiogenic shock, 3 (1.29%) cases of various arrhythmia, 4 (1.72%) cases of late revascularization, and 3 (1.29%) cases of heart failure rehospitalization. Among the 178 patients with CIN(−), 2 experienced a MACE, which included 1 (0.43%) case of non-fatal myocardial infarction and 1 (0.43%) case of late revascularization. Left main disease and three-vessel disease were characteristic of those who experienced a MACE. The percentage of MACE incidence among CIN(+) patients was significantly higher than in the CIN(−) group (24.07% vs. 1.12%, *p *< 0.001). The predictive abilities of biomarker combinations for MACE occurrence rate were assessed (Table 6; Figure 7). The AUC-ROC values of uNAG and sHCY for detecting MACE incidence rate were 0.637 and 0.826, respectively. However, as shown in Figure 7, the AUC-ROC value of their combinations in predicting MACE incidence rate indicated no significant advantage over that of single sHCY. The combined detection did not enhance the predictive capability for a MACE in patients with CIN(+).

## 4 Discussion

Our main finding of the present observational study was that the panel of uNAG plus sHCY demonstrated superior discriminative performance in CIN detection when compared to the single biomarker. It also provided relevant clinical prognostic information about CIN. To our knowledge, the present study was the first to show that a panel of uNAG plus sHCY yielded greater predictions for CIN in an adult general coronary intervention cohort after contrast agent exposure.

Due to the considerable advancements in pharmacological therapy and interventional procedures, a growing number of patients with CAD are currently revascularized percutaneously, especially in cases of confirmed or suspected acute coronary syndromes (17). There is an increasing proportion of high-risk patients, including those with impaired renal function (18). In fact, some previous studies have reported that the incidence of CIN is up to 23.7%–30% (19, 20). Although pretreatment strategies such as hydration and acetylcysteine administration have been identified, the incidence of CIN remains high. Identifying and further reducing the high risk for patients who undergo such procedures and develop complications is of extreme importance. In 2004, Maeder et al. proposed a CIN risk stratification score based on eight readily available variables to better identify patients at high risk of CIN (21).

Several potentially valuable serum and urine biomarkers of kidney injury have been identified, which play a warning role in predicting kidney injury at an early stage in clinical practices, such as NAG, CysC (9, 22), neutrophil gelatinase-associated lipocalin (NGAL) (23), homocysteine (24), urinary microalbumin (25), tissue inhibitor of metalloproteinase 2, and insulin-like growth factor-binding protein 7 (26). In the field of coronary intervention, contrast media-associated acute kidney injury (CA-AKI) has become another major challenge for cardiologists following restenosis and stent thrombosis. The lack of early, specific, and sensitive biomarkers leads to delayed early diagnosis and intervention of CIN, which is an important reason for the high mortality rate among patients with CIN following coronary intervention. Therefore, effective biomarkers or their combinations for early renal function impairment stage in CIN detection are key factors in preventing this complex complication.

NAG is an acidic hydrolase. Since the large size of the uNAG molecule impedes its renal filtration, high levels of uNAG are unlikely to originate from a non-renal source (27). Serving as a tubular damage biomarker, it can directly reflect the damage to proximal convoluted tubules and is positively correlated with renal function damage. A number of studies have confirmed that uNAG is an indicator with good sensitivity and specificity for early diagnosis of CIN (22) and is closely associated with mortality (9). Homocysteine is a cytotoxic sulfur-containing amino acid metabolized primarily by the kidney. Enzymes related to sHCY metabolism are all present in the kidney. The deficiency or loss of activity of these enzymes may result in the blockage of metabolic pathways when renal function is impaired, thereby causing the accumulation of sHCY in the body, leading to glomerular sclerosis and injury (28). Serum homocysteine concentrations have been identified to be clearly correlated with renal function. Elevated levels of homocysteine invites endothelial dysfunction, proliferation of vascular smooth muscle cells, decreased bioavailability of nitric oxide, and the generation of free radicals and oxidative stress (11). In addition, a high concentration of homocysteine can cause vascular damage and alter the coagulation process, which is associated with an elevated risk of atherosclerosis and vascular injury. It also contributes to further renal damage through intense vasoconstriction and the loss of autoregulatory capacity, both of which are mediated by the release of reactive oxygen species. Animal experimental models (29) and multiple clinical studies (30) have shown that sHCY level is an independent risk factor for CIN. A study shows that elevated homocysteine levels are associated with an increased risk of CIN in patients undergoing PCI (10). Other studies have indicated that exposure of kidney tissue to highly permeable contrast agents can lead to direct tubular toxicity, presenting as acute tubular necrosis. By stimulating the release of potent vasoconstrictors such as endothelin and adenosine, the iodine contrast medium can immediately constrict blood vessels and exert an impact on hemodynamic changes in the kidney, potentially resulting in renal impairment. Because the effects of hyperhomocysteinemia on vascular function are similar to the pathophysiological mechanisms of contrast nephropathy, it is valuable to investigate the relationship between homocysteine levels and the development of CIN.

The pathogenesis of CIN results from endothelial dysfunction, cellular toxicity caused by the contrast agent, and tubular apoptosis resulting from hypoxic damage or reactive oxygen species. In fact, most studies thus far have focused on the individual abilities of biomarkers to detect CIN in coronary intervention patients, but a single biomarker is not sensitive or specific enough to reflect the multiple pathophysiologies of CIN. Researchers in several past studies have discovered the superiority of various combinations of biomarkers, reporting increased predictive performance for AKI with the different combinations they employed (31). However, few researchers have studied the combination of biomarkers for CIN occurrence. Although the combination of the inflammatory biomarkers hypersensitive C-reactive protein (Hs-CRP) and procalcitonin have been used as a predictor of CIN (32), the potential predictive abilities of a combination representing both tubular damage and cellular toxicity biomarkers have not yet been completely examined in adult coronary intervention patients. A combination reflecting underlying the pathological processes of CIN may be a reasonable strategy and superior to individual biomarkers alone. In our research, the combination of uNAG and sHCY was assessed for CIN detection and clinical prognosis.

In the present study, the incidence of CIN was 23.28%, which is consistent with the previous studies (19). The incidence of CIN in patients with cerebrovascular disease and renal insufficiency diseases was significantly higher than that in the non-CIN group (*p *< 0.05). The result may be attributed to some pathogenic factors such as blockage of small arteries stemming from atherosclerosis and aging, leading to endothelial damage, tubular inflammation, and activation of intrarenal fibrotic pathways. Long-term treatment with multiple medications for chronic cerebrovascular disease can be nephrotoxic. In addition, it is hypothesized that brain damage may exert a detrimental effect on the nephron, which is called brain-renal syndrome. This finding is very similar to those reported in meta-analyses by Zorrilla-Vaca (33). Moreover, direct renal injury, reactive oxygen species, and activation of the sympathetic nervous system play a critical role in the development of CIN, and beta-blockers are frequently used as a common preventive strategy to reduce sympathetic nervous activity (34). This may be potentially illustrated by the fact that patients taking beta-blockers had a lower rate of CIN in our study. It has been reported that CA-AKI is significantly associated with higher GRACE scores and STEMI. Acute myocardial infarction (AMI) causes the release of many active metabolites, leading to hemodynamic and inflammatory changes that deteriorate renal blood flow (35, 36). Patients who developed CIN had higher triglyceride (TC) and LDL-C levels, and high CRP levels compared to those who did not develop CIN (37). A study emphasized the increasing role of invasive procedures (cardiac catheterization and cardiovascular surgery) and fibrinolysis/anticoagulation as triggering factors for CIN. Catheter manipulations can disrupt atherosclerotic aortic plaques, exposing the soft, cholesterol-laden core of the plaque to the arterial circulation. Anticoagulant or thrombolytic treatment may initiate the disruption of complex plaques by causing internal hemorrhage or lysis of intraintimal or cap thrombi. Abrupt or repeated rupture of unstable plaques, a massive shower of emboli, or multiple cyclic embolizations might represent a possible cause for the development of CIN (38). According to research, the onset of CIN may be related to the production of inflammatory cytokines and chemokines in the kidneys, the upregulation of leukocyte adhesion factor, and the infiltration of various inflammatory cells into the kidneys. Serving as an indicator of inflammation, CRP is also involved in the inflammatory response, leading to an increased risk of CIN. Therefore, the interaction of inflammatory responses, oxidative stress, and hypoxia of the medulla mutually aggravate the renal toxicity induced by contrast media and contribute to the pathogenesis of CIN (32). All of the above findings are consistent with ours. In addition, the correlation between the incidence of CIN and the duration of PCI may be attributed to more severe arteriosclerosis and complicated vascular disease in patients, such as chronic total occlusion (CTO). Some of our study's results are inconsistent with those of previous studies (39). For instance, factors such as advanced age, male gender, diabetes mellitus (DM), systemic arterial hypertension, Mehran score, and the volume of contrast infused may be related to the number of cases or composition of patients in the study.

uNAG and sHCY have both manifested well as an early damage biomarker for CIN and can also predict poor outcomes, respectively. In fact, the predictive abilities of sHCY and uNAG for CIN detection and prognosis have varied across different published studies (11, 22). When NAG was calculated at different time periods and in various cases, it exhibited different manifestations for CIN detection (8, 39). The sensitivity for CIN detection was higher than its specificity, especially within 24 h after PCI (*T2*–*T4*). sHCY exhibited a similar performance. The sensitivity of sHCY for CIN detection was significantly higher than its specificity during the perioperative period of PCI and before discharge (*T1*–*T6*). Meanwhile, the sensitivity of sHCY for CIN detection was relatively higher than that of NAG. Within 24 h after PCI (*T2*–*T4*), the specificity of sHCY in detecting CIN was relatively higher than that of NAG. Moreover, the combination of uNAG and sHCY demonstrated the highest sensitivity at 12 h (*T3*) and the highest specificity at 24 h (*T4*) after PCI. But as a single biomarker, uNAG and sHCY had the highest specificity and sensitivity at 48 h (*T5*) respectively, both of which were similar later when used in their combinations, suggesting the advantages of combined biomarkers over single biomarkers for early CIN detection. In addition, the specificity of sHCY for MACE prediction was much greater than that of uNAG, as its sensitivity was limited. For MACE prediction, the sensitivity of the combined biomarkers was higher than their specificity and the sensitivity as a single biomarker. In our study, uNAG showed poor to moderate AUC-ROC values for CIN detection and MACE prediction, while sHCY demonstrated significantly higher AUC-ROC values than that of uNAG, indicating that sHCY has a moderate or more powerful predictive ability. The AUC-ROC values of the biomarker combinations at 12 h after PCI (*T3*) exceeded 0.8, suggesting that the combination of uNAG and sHCY showed superior predictive ability for CIN prediction earlier than the diagnosis based on sCr levels. Furthermore, the combination of uNAG and sHCY yielded greater AUC-ROC values (0.832) for MACE prediction, indicating stronger performance in predicting adverse clinic outcomes, but there was no prominent advantage compared with the AUC-ROC value of single sHCY.

In a stratified study of CIN severity, sCr had the ability to distinguish between mild and severe CIN to some extent. Although biomarkers had great predictive ability for the detection of CIN, their abilities to distinguish between mild and severe CIN were limited. In our study, sHCY served as a cellular toxicity biomarker with high sensitivity for CIN detection, while uNAG functioned as a kidney tubular damage biomarker with relatively higher specificity, its increasing level was earlier than that of sCr (*T5*). The combination of sHCY and uNAG yielded greater clinical diagnostic performance in detecting CIN and providing relevant prognostic information. This could be instrumental in making their respective advantages complementary to each other. This combination's advantage may be put down to the fact that it consists of a cellular toxicity biomarker with high sensitivity and a renal tubular damage biomarker with high specificity, reflecting potential nephron-damaging mechanisms in the generation of CIN. This study suggests that combining specimens from different sources (urine and serum) with different characteristics is reasonable as they provide a superior biomarker panel for the diagnosis and prognosis of CIN in complicated and volatile clinical situations. Our conclusions are consistent with those of previous studies (32, 39). This approach is also in line with the mainstream research direction of the Acute Dialysis Quality Initiative (ADQI), which recommends combining two or more biomarkers in the prediction of CA-AKI to improve its efficiency and accuracy (40).

It is worth noting that our study has its limitations. First, the specificity of the combination of these biomarkers in predicting CIN was relatively low compared to other studies (11, 39) and there was no significant difference between the CIN group and non-CIN group in terms of length of CCU stay, length of hospital stay, renal replacement therapy, CCU mortality, and in-hospital mortality (*p* > 0.05). Furthermore, the study had relatively small sample size which may have blunted the statistical effectiveness of the results. Moreover, some patients with coronary interventional surgery were not admitted in time, causing the urine samples to be affected by drugs and organism volumetric status, or kidney replacement therapy was not performed promptly. We also failed to compare the selected biomarkers with other commonly used clinical biomarkers or their combinations, and we did not consider the effects of different medical interventions on patient outcomes during cardiovascular hospitalization. Therefore, a large sample, multicenter, observational trial is needed to further determine the value of uNAG and sHCY for predicting the risk of CIN, to improve the accuracy and timeliness of CIN diagnosis and establish better interventional measures to improve prognosis.

## 5 Conclusion

The present study shows that the combination of a tubular damage marker (uNAG) and a cellular toxicity marker (sHCY) in Cardiovascular Medicine Wards has significantly better discriminative performance for CIN detection than the individual biomarkers. This study was conducted in general adult Cardiovascular Medicine Wards with an observational cohort. Therefore, our results could have significant clinical implications for patients who underwent percutaneous coronary intervention at risk for CIN.

## Data Availability

The original contributions presented in the study are included in the article/Supplementary Material, further inquiries can be directed to the corresponding author.

## Glossary

CIN: contrast-induced nephropathy
PCI: percutaneous coronary intervention
uNAG: urine N-Acetyl-β-D-glucosaminidase
sHCY: serum homocysteine
sCr: serum creatinine
ROC: receiver operating characteristic curves
AUC: area under the curve
CCU: Coronary Heart Disease Intensive Care Unit
MACE: major adverse cardiovascular events
ESRD: end-stage renal disease
eGFR: estimated glomerular filtration rate
GRACE: Global Registry of Acute Cardiovascular Events
BMI: body mass index
DM: diabetes mellitus
HF: heart failure
COPD: chronic obstructive pulmonary disease
ACEI: angiotensin-converting enzyme inhibitor I
ARB: angiotensin II receptor antagonist
CCB: calcium channel blocker
RRT: renal replacement therapy
NT-proBNP: N-terminal pro B-type natriuretic peptide
Hb: hemoglobin
HCT: hematocrit
RDW-CV: variation coefficient of erythrocyte volume distribution width
RDW-SD: standard deviation of erythrocyte distribution width
CRP: C-reactive protein
Hs-CRP: hypersensitive C-reactive protein
ALB: albumin
CHO: total cholesterol
TC: triglyceride
HDL-C: high-density lipoprotein
LDL-C: low-density lipoprotein
Lpa: lipoprotein a
HbA1c: glycosylated hemoglobin
T3: triiodothyronine
T4: thyroxine
FT3: free triiodothyronine
FT4: free thyroxine
TSH: thyroid stimulating hormone
STEMI: ST-segment elevation myocardial infarction
NSTEMI: non-ST-segment elevation myocardial infarction
ACS: acute coronary syndrome
UA: unstable angina
CAD: coronary artery disease
*T1*: pre-PCI,
*T2*: within 1 h after PCI
*T3*: 12 h after PCI
*T4*: 24 h after PCI
*T5*: 48 h after PCI
*T6*: before discharge
CI: confidence interval
PPV: positive predictive values
NPV: negative predictive values
[+]LR: positive likelihood ratios
[−]LR: negative likelihood ratios
